# The impact of hypertension prevention and modification on dementia burden: A systematic review of economic studies

**DOI:** 10.1016/j.tjpad.2024.100017

**Published:** 2025-01-01

**Authors:** Marie Lan, Ava John-Baptiste, Cassandra Curran, Feben W. Alemu, Abolfazl Avan, Kelly K. Anderson, Shehzad Ali

**Affiliations:** a Epidemiology and Biostatistics, Schulich School of Medicine & Dentistry, Western University, London, ON, Canada; b London Health Sciences Centre, London, ON, Canada; c Lawson Health Research Institute, London, ON, Canada; d Schulich Interfaculty Program in Public Health, Western University, London, ON, Canada; e Department of Anesthesia and Perioperative Medicine, Western University, London, ON, Canada; f Centre for Medical Evidence, Decision Integrity and Clinical Impact, London, ON, Canada; g Robarts Research Institute, University of Western Ontario, London, Ontario, Canada; h Psychiatry, Schulich School of Medicine & Dentistry, Western University, London, ON, Canada; i WHO Collaborating Centre for Knowledge Translation and Health Technology Assessment in Health Equity, Ottawa, Canada; j Department of Health Sciences, University of York, York, UK; k Centre for Addiction and Mental Health, Institute for Mental Health Policy Research, Toronto, Ontario, Canada; l Department of Psychology, Macquarie University, Sydney, Australia

**Keywords:** Dementia, Costs, Economic evaluation, Hypertension, Prevention

## Abstract

**Aim:**

Neurological disorders account for the largest proportion of disability-adjusted life years globally, with dementia being the third leading cause. Hypertension has been identified as a priority, targetable risk factor for dementia. This study aimed to systematically review economic studies that examine the impact of hypertension prevention and control on the costs and outcomes of dementia.

**Methods:**

An electronic literature search was conducted using MEDLINE, EMBASE, Scopus, Web of Science, EconLit, and grey literature sources. The inclusion criteria were: 1) economic evaluation studies, including both full and partial evaluations; 2) a primary focus on dementia; and 3) evaluation of the impact of preventing or modifying hypertension on the burden of dementia. The quality of included studies was assessed using the Consensus on Health Economic Criteria (CHEC) list.

**Results:**

Twelve studies were included in the final review. Four studies were full economic evaluations, while eight were partial evaluations, with one reporting costs and seven reporting the impact on dementia prevalence. Nine studies considered hypothetical reductions in hypertension rate, while three evaluated applied hypertension-related interventions. Hypertension modification was associated with higher life expectancy and a higher average age of dementia onset. Full economic evaluations of specific hypertension modification interventions found that these interventions dominated (i.e. had lower costs and higher quality-adjusted life-years (QALY)) the status quo scenario or had an acceptable incremental cost-effectiveness ratio (ICER).

**Conclusions:**

Hypertension modification has the potential to reduce the burden of dementia in a cost-effective way. However, further economic evaluations of applied interventions are needed to determine real-world feasibility and cost-effectiveness.

## 1 Introduction

Neurological disorders account for the largest proportion of disability-adjusted life years globally (10.8%), with dementia being the third leading cause after stroke and migraine. [1,2] It is estimated that the number of people with dementia will increase from 57.4 million in 2019 to 152.8 million by 2050 globally. [3] The estimated global economic burden of dementia was US$1.313 trillion in 2019. [4]

The World Health Organization's global action plan 2017–2025 highlighted dementia as a public health priority and called for global action in the area of dementia risk reduction. [5] A major modifiable risk factor for vascular dementia, which accounts for 17–30% [6] of total dementia cases, is hypertension. Hypertension increases the risk of stroke which in turn doubles the risk of developing dementia (RR: 2.18, 95%CI: 1.90–2.50). [7] The Ontario Stroke Strategy which was aimed at treating and preventing stroke found that a 32% decrease in the incidence of stroke was associated with a 7% decrease in dementia at the population level. [8] Hypertension is also associated with cognitive decline independent of stroke, with those with hypertension and no stroke history having almost twice the risk of vascular dementia. [9]

Midlife hypertension has been identified as a priority targetable risk factor for dementia alongside physical inactivity, obesity, diet, tobacco use, alcohol use, and diabetes. [5] Recent systematic reviews and meta-analyses found that lower blood pressure and the use of anti-hypertensive medication reduced dementia risk by 21% (RR: 0.79, 95%CI: 0.70–0.89) [10] and led to improved cognition (SMD: −0.049, 95%CI: −0.078, −0.019). [11] Worldwide, hypertension affects more than 30% of adults. [12] It is estimated that 46% of adults with hypertension are unaware that they have the condition, and only 21% of adults with hypertension have it under control. [13] The high lifetime risk of hypertension combined with the low likelihood of hypertension control despite the availability of treatments makes it a readily targetable risk factor for dementia prevention.

While the relationship between hypertension and dementia is well studied, there has not been a systematic review of the economic impact of hypertension modification interventions on dementia burden. This economic evidence is imperative for resource allocation decisions and the implementation of wide-scale prevention and management programs. Therefore, the objective of this study was to conduct a systematic review of economic studies investigating the impact of hypertension modification on the costs and health outcomes related to dementia.

## 2 Methods

The present study was registered through PROSERO (CRD42023478744) and was conducted in accordance with the PRISMA 2020 reporting guidelines for systematic reviews.

### 2.1 Search strategy

An electronic literature search of MEDLINE, EMBASE, Scopus, Web of Science, EconLit, RePEC, NHS Economic Evaluation Database (NHS EED), and Cochrane Library was conducted on October 13, 2023. Grey literature sources that were also searched include: the International Network of Agencies for Health Technology Assessment (INAHTA) database, OECDi Library, Alzheimer's association, and Alzheimer's disease international.

A comprehensive search strategy was developed and tested to ensure that relevant studies were identified. To do so, well-established search strategies for economic studies produced by the Canada Drug Agency (CDA) were used. [14,15] The following keywords and related terms were used for the search (CDA Search filter or “Health Care Economics and Organizations” or “economic evaluation” or “health economics”) AND (“dementia” or “Alzheimer's” or “cognitive impairment”) AND (“hypertension” or “blood pressure”). There were no restrictions on country, language or publication period. Searches were adapted to each database appropriately and were set to notify authors of new relevant articles each month. When new articles were added to the searches, two reviewers (ML and CC) reviewed them. None of the additional studies met the inclusion criteria. References of included studies were also searched. The detailed search strategies for each database are outlined in the appendix.

### 2.2 Selection criteria

Records were imported into Covidence, and duplicates were removed. Eligibility was assessed by two independent reviewers (ML and CC) in two stages: first, title and abstracts were reviewed to screen for relevant studies, and then full-text articles were retrieved and reviewed. Inclusion criteria were: 1) economic evaluation study, including full and partial evaluations; 2) primary focus on dementia risk; and 3) evaluated the economic impact of preventing or modifying hypertension on the burden of dementia. Full economic evaluations were defined as studies assessing changes in both costs and health outcomes. Partial economic evaluations, in contrast, examined changes in either costs or outcomes, but not both. Reasons for exclusion were documented at the full-text screening stage. Discrepancies between the reviewers were resolved by an independent third reviewer (FWA).

### 2.3 Data collection and quality assessment

Data from the included studies were manually extracted into a Microsoft Excel spreadsheet. The characteristics of the study (authors, publication year, title, country, study type, study population), methodological information (intervention, comparator, perspective, data sources, model inputs, time horizon, discounting, sensitivity analyses), and results (incremental costs and effects, net benefits, prevalence estimates, cases avoided, lifetime risk for dementia, probability of being cost-effective) were documented. Data were extracted by two independent reviewers (ML and CC), with a third reviewer resolving discrepancies (FWA).

The quality of the studies was assessed using the Consensus on Health Economic Criteria (CHEC)- Extended list [16] which consists of 20 binary questions (Appendix A.2). Criteria that were not applicable were marked as “no”. The quality appraisal was completed by two independent reviewers (ML and CC), with discrepancies being resolved by a third independent reviewer (FWA).

Due to the heterogeneity of studies and settings, a narrative synthesis was used to summarise the findings. Cost values were converted to 2024 international dollars (Int'l$) using region-specific inflation rates [17] and OECD purchasing power parities [18] to aid in comparisons. Studies that did not report the currency year were assumed to have the same currency year as the publication.

## 3 Results

**Fig. 1:**
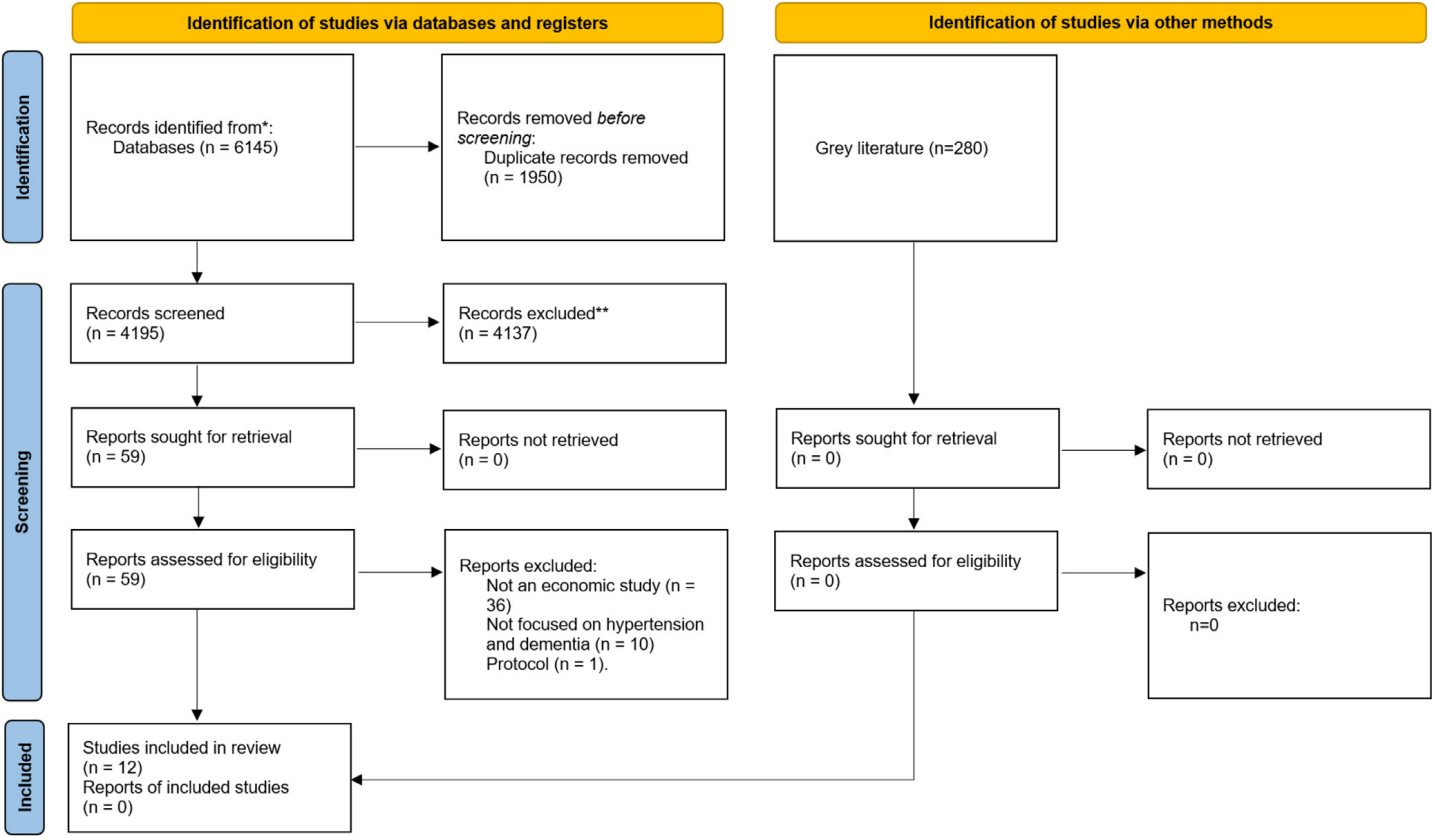
PRISMA Diagram.

The search strategy retrieved 6145 records from databases and 280 from grey literature sources (Fig. 1). After duplicates were removed, 4475 studies were screened. There were 59 articles eligible for full-text screening, of which 12 were included in the final review. Studies were excluded at the full-text level for the following reasons: not an economic study (*n* = 36), does not evaluate the impact of modifying or preventing hypertension (*n* = 10), protocol (*n* = 1).

### 3.1 Characteristics of included studies

Four of the included studies were full economic evaluations (Table 1), while eight were partial economic evaluations (Table 2**);** one evaluated costs only [27] and seven evaluating outcomes (ex. changes in dementia prevalence, cases avoided, etc.) only. [[23], [24], [25], [26],[28], [29], [30]] 11 studies were published in English while one was published in German [28]. The studies were published between 2011 and 2023 and originated from the United Kingdom (*n* = 2), France (*n* = 2), United States (*n* = 2), Germany (*n* = 1), Australia (*n* = 1), Spain (*n* = 1), and Sweden (*n* = 1). Two of the studies had a global perspective [23,29]: one had an additional sub-analysis for the United States only [23], while the other had sub-analyses for the United States, Europe, and the United Kingdom. [29]

**Table 1:** Characteristics of Full Economic Evaluations (*n* = 4).

Study	Country	Economic Evaluation Type	Population Characteristics	Describe intervention	Comparator	Perspective	Cycle Length	Time Horizon	Effectiveness Measure	Outcome	Discounting Rate	Sensitivity Analyses
**McRae 2021 [**19**]**	Australia	Model-Based CEA	45 and older, average age 65 years. 65+ expected to develop dementia	5% improvement in risk factors (midlife obesity, physical inactivity, smoking, low educational attainment, diabetes mellitus, midlife hypertension, depression) (Hypothetical)	No intervention	Societal	NR	Lifetime	QALY, Maximum program cost	**Maximum program cost to be cost saving:** Int'l$156 ($189AUD) **Maximum program cost to be cost-effective** (WTP= Int'l$41,358 ($50,000AUD): Int'l$381 ($460AUD).	3%	Deterministic sensitivity analysis
**Mukadam 2020 [**20**]**	United Kingdom (England)	Model-Based CUA	English population; Aged 45–100 years, closed cohort	Target those with untreated and uncontrolled midlife hypertension with healthcare visits and medication (Hypothetical)	No intervention	Societal	1 Year	Lifetime, 55 cycles,	QALY	**Prevalence:** 5% reduction in dementia prevalence **ICER:** Int'l$ 21,720 (£9555)/QALY.	3.5%	Deterministic and probabilistic sensitivity analyses
**Soto-Gordoa 2015 [**21**]**	Spain	Model-Based CEA	40 years or older, open cohort (new cohort of 40-year-olds entering each year)	% and 20% reduction in hypertension prevalence (Hypothetical)	No intervention	Societal	1 Year	2010 to 2050	Cases Prevented	**10% reduction:** Cases prevented: 13,511 Cost Savings: Int'l$1.231billion (648 million Euros) **20% reduction:** Cases prevented: 26,674. Cost Savings: Int'l$2.429 billion (1.279 billion Euros).	None	None
**Zhang 2011 [**22**]**	Sweden and Finland	Model-Based CUA	Aged 50–70 years.	Health promotion program and pharmacological treatment of cardiovascular risk factors (hypertension, hyperlipidemia, diabetes). (Hypothetical)	No intervention	Societal	1 Year	20 years	QALY	**ICER**: Dominated - intervention is cheaper and better. **Probability of being cost-effective** (WTP: Int'l$107,567; 600,000 SEK): 67%	3% (0%−5% in sensitivity)	Deterministic and probabilistic sensitivity analyses

*UC: Usual Care/no intervention; QALY: Quality-adjusted life-year; ICER: incremental cost-effectiveness ratio; WTP: willingness-to-pay.

**Table 2:** Characteristics of Partial Economic Evaluations (*n* = 8).

Author year	Country	Approach	Population Characteristics	Describe Intervention	Comparator	Time Horizon	Outcome	Sensitivity Analyses
Barnes and Yaffe 2011 [23]	Global, United States	Non-simulation-based	General overall population	10% or reduction in hypertension prevalence (hypothetical)	No intervention	Unclear	**World:** 10% lower: ∼160,000 fewer cases 25% lower: ∼400,000 fewer cases **USA:** 10% lower: ∼40,000 fewer cases 25% lower: ∼100,000 fewer cases	None
Chen 2023 [24]	United Kingdom (England and Wales)	Markov Model	Aged 35 to 100, healthy, open population	50% decrease in hypertension prevalence by 2060 (hypothetical)	No intervention	2017 to 2060	**Dementia Prevalence difference**: +24.5 thousand (−37.5 to −12.7 thousand). **Cases avoided:** −9.0 (−13.2 to −5.1) per 100,000 by 2060 **Life expectancy:** +8% (5.3 vs 4.9 years) at age 65	Yes, changing mortality rates.
Jacqmin-Gadda 2013 [25]	France	Markov Model	Age 65 at start, open population	1) 50% reduction of hypertension prevalence at age 65 (hypothetical) 2) 100% reduction of hypertension prevalence at age 65 (hypothetical) 3) Antihypertensive drug that reduces the risk of dementia by 15% and death by 43% (hypothetical)	No intervention	2010 to 2030	**Dementia Prevalence changes:** UC=872,000 1) −0.7% 2) −1.4% 3) +5.6%	Yes, changing mortality rates.
Jacqmin-Gadda 2023 [26]	France	Simulation-based	Aged 45 to 85 at start, open population	Complete disappearance of hypertension inactivity (hypothetical)	No intervention	2020 to 2040	*Difference in:* **Prevalence:** −3.14% for men, −3.63% for women **Lifetime risk for dementia**: −5.43% for men, −2.98% for women **Overall life expectancy:** +1.52 years for men, +0.96 years for women **Age at dementia onset:** +1.4 years for men, +0.94 for women	None
Lin 2014 [27]	United States	Simulation-based	Aged 65 years at start, closed cohort	Reduction of risk factor by 10% (hypothetical)	No intervention	Lifetime	*Lifetime direct medical costs per capita:* **Intervention:** Int'l$29,270 ($21,433) **UC:** Int'l$32,064 ($23,471) **Net:** -Int'l$3578 (-$2620)	None
Luck 2016 [28]	Germany	Non-simulation-based	General German Population	Reduction of high blood pressure by 10%, 25%, 50% (hypothetical)	No intervention	Unclear	**Cases avoided** (based on estimated prevalence of 1 million cases): 10% reduction: 7023 cases 20% reduction: 17,405 cases 50% reduction: 36,336 cases	None
Norton 2014 [29]	Global, United Sttes, Europe, United Kingdom	Non-simulation-based	General overall population	10% or 20% reduction in all risk factors per decade (hypothetical)	No intervention	2010 to 2050	**Cases Avoided (10%, 20% reduction):** World: 8·3% (8·8 million); 15·3% (16.2 million) USA:8·7% (0·8 million); 16·3% (1·5 million) Europe: 9·1% (1·5 million); 16·9% (2·8 million) UK: 8·8% (0·2 million); 16·2% (0·3 million)	None
Zissimopoulos 2018 [30]	United States	Markov Model	51 years+, closed cohort	1) Reduce incident hypertension by 50% (hypothetical) 2) No incident hypertension (hypothetical) 3) Complete elimination of existing hypertension and no incident hypertension (hypothetical)	No intervention	2010 to 2040	*Differences in:* **Life expectancy (years),** UC=21.48 years: 1) +0.36; 2) +0.53; 3) +0.87 **Lifetime dementia risk, UC=34.7%** 1) +0.7%; 2) +2.1%; 3) +3.6%	None

*UC: Usual Care/no intervention.

### 3.2 Appraisal of full economic evaluations

#### 3.2.1 Model design and population

All four full economic evaluations were model-based cost-effectiveness analyses. All models had a starting age during midlife, ranging from age 40–50 years. Mukadam et al. (2020) and Zhang et al. (2011) evaluated the treatment of cardiovascular risk factors using Markov models which consisted of 55 cycles and 20 cycles respectively with cycle lengths of 1 year. Mukadam et al. (2020) modeled a cohort from age 45 to 100 years, while Zhang et al. (2011) modeled a cohort from age 50 to 70 years. Soto-Gordoa et al. (2015) modeled a 40-year-old or older population from 2010 to 2050, with a cycle length of 1 year with a new 40-year-old cohort entering each cycle. McRae et al. (2021) used a novel method to estimate the maximum cost per person for a dementia prevention program to be cost saving. They used the estimated number of dementia cases prevented through reducing cardiovascular risk factors by 5%, the expected quality-adjusted life years (QALYs) saved, the lifetime cost of dementia per person, and a willingness to pay (WTP) of Int'l$41,358 ($50,000AUD) to inform the maximum program cost. Two studies derived dementia status based on the CAIDE Dementia Risk Score, [21,22] while two used region-specific relative risks [19,20] which were derived from various large-scale observational studies.

Three of the studies had discounting, with rates of either 3% [19,22] or 3.5% [20], with one study conducting sensitivity analysis on the discount rate between 0% and 5% [22], and another varying the discount rate between 3% and 5%. [19] Three of the four studies conducted sensitivity analyses. Mukadam et al. (2020) and Zhang et al. (2011) completed one-way deterministic sensitivity analysis and probabilistic sensitivity analyses using Monte-Carlo simulation. McRae et al. (2021) conducted deterministic sensitivity analysis only.

#### 3.2.2 Hypertension scenarios investigated

The types of hypertension scenarios assessed in the full economic evaluations varied. McRae et al. (2021) assessed the effect of decreasing a set of risk factors (midlife obesity, physical inactivity, smoking, low educational attainment, diabetes mellitus, midlife hypertension, depression) uniformly by 5% over the course of 20 years. Mukadam et al. (2020) evaluated interventions for several dementia risk factors (smoking, diabetes, hearing loss), and one for hypertension which hypothetically treated 22% of the mid-life population with untreated or uncontrolled hypertension in the population. Soto-Gordoa et al. (2015) assessed the impact of a 10% or 20% reduction in hypertension. Lastly, Zhang et al. (2011) evaluated the impact of a health promotion program in tandem with a pharmacological treatment of cardiovascular risk factors (hypertension, hyperlipidemia, and diabetes). The intervention assumed a 40% prevalence of hypertension at age 50, with 50% of people treated. The comparator for all studies was usual care/no intervention.

#### 3.2.3 Perspective of the economic evaluations

All studies used a societal perspective, considering direct, indirect, formal, and informal costs. All studies used costs based on service use data collected in national government and administrative data sources or data from large-scale observational studies. Mukadam et al. (2020) and Zhang et al. (2011) used annual costs for each disease state to estimate total costs, while McRae et al. (2021) used lifetime dementia cost estimate as a model input. Soto-Gordoa et al. (2015) modelled cases prevented and multiplied it by per capita dementia costs to estimate overall cost savings.

Three of the studies used region-specific quality-adjusted life year (QALY) to measure effectiveness. [19,72,^22^] McRae et al. (2021) and Zhang et al. (2011) used QALY values derived from the EQ-5D questionnaire. Mukadam et al. (2020) used EQ-5D-based utility values for mild and moderate dementia but HUI Mark II values for severe dementia. Soto-Gordoa et al. (2015) assessed intervention effectiveness by reporting the number of cases prevented. None of the studies considered caregiver disutility (ie. quality of life impacts associated with caregiving).

#### 3.2.4 Findings of full economic evaluations

Overall, the full economic evaluations indicated that hypertension modification can potentially be a cost-effective mechanism for decreasing the burden of dementia. McRae et al. (2021) modeled the reduction in the prevalence of hypertension and other cardiovascular risk factors uniformly by 5% and estimated a 3.2% reduction in dementia burden using population attributable fractions. The authors identified that the maximum cost per person at which a risk-factor prevention program would still cost-effective was Int'l$381 ($460AUD), while the maximum cost for a program to be cost-saving was Int'l$156 ($189AUD), under an Int'l$41,358 ($50,000AUD) per QALY WTP.

Soto-Gordoa et al. (2015) projected that a 10% reduction in hypertension in 2010 would lead to 13,511 cases of dementia prevented in 2050, leading to savings of Int'l$1230,632,409 (648 million euros). A 20% reduction in hypertension in 2010 would lead to 26,674 cases of dementia prevented in 2050 with expected savings of Int'l$2428,979,708 (1.279 billion euros).

The antihypertensive therapy in Mukadam et al. (2020) had a lifetime gain of 0.0393 QALYS, and cost of Int'l$854 (£376), leading to an incremental cost effectiveness ratio (ICER) of Int'l$ 21,720 (£9555) per QALY. The intervention led to a 5% reduction in the prevalence of dementia and had expected net annual savings of Int'l$2.448 billion (£1.077 billion). The antihypertensive intervention had an 85% probability of being cost effective at an Int'l$45,465 (£20,000) threshold, and a 97% probability of being cost effective at an Int'l$68,197 (£30,000) threshold.

The combination treatment in Zhang et al. (2011) led to cost reduction of Int'l$3939 (21,974SEK), and an increase of 0.0511 QALYs compared to usual care, resulting in the intervention dominating the usual care. With a WTP of Int'l$107,567 (600,000SEK), the probability of being cost effective was 67%.

All full economic evaluations were conducted from the perspectives of specific countries, limiting the ability to compare by region. None of the full economic evaluations reported sex differences.

### 3.3 Appraisal of partial economic evaluations

#### 3.3.1 Model design and population

The partial economic evaluations primarily consisted of papers that projected changes in the future prevalence of dementia due to hypothetical reductions in hypertension prevalence. Of the eight articles identified, one evaluated cost only, [27] while the remaining studies only evaluated the prevalence of dementia. [[23], [24], [25], [26],[28], [29], [30]] Three studies used Markov models [24,25,30], two used other simulation approaches [25,27] and three used non-simulation-based approaches [23,28,29]. The time horizons for the studies varied between 20 years to 40 years. None of the studies used discounting. Only two studies included sensitivity analyses that included adjustments for mortality projections. [24,25]

The minimum age of the study populations varied between 35 years and 65 years. Chen et al. (2023) modeled an open population aged 35–100 years from 2017 to 2060. Jacqmin-Gadda et al. (2013) modeled an open cohort of 65-year-olds from 2010 to 2030, while Jacqmin-Gadda et al. (2023), modeled an open cohort of 45–65-year-olds from 2020 to 2040. Lin et al. (2014) and Zissimopoulos et al. (2018) modeled closed cohorts for their lifetime, beginning at age 65 and 51 years respectively. Barnes and Yaffe (2011), Luck et al. (2016), and Norton et al. (2014) assessed dementia prevalence at the population level but did not specify the specific target population. Barnes and Yaffe (2011) and Luck et al. (2016) evaluated the number of current cases that could be avoided with hypertension prevalence reduction whereas Norton et al. (2014) modeled results from 2010 to 2050.

Three studies focused on Alzheimer's disease while the remaining studies considered all dementias. Estimates of dementia incidence and prevalence were derived from studies based on diagnostic measures such as cognitive tests, ICD codes, report of a doctor diagnosis, or use of certain medication (*n* = 5) [[24], [25], [26], [27],^30^] or through population-wide estimates (*n* = 3). [23,28,29]

All studies used large-scale observational studies for dementia prevalence or risk estimates. Barnes and Yaffe (2011) used pooled population-level estimates. Lin et al. (2014) used administrative data to determine dementia cost estimates. Luck et al. (2016) and Norton et al. (2014) used data from the Health Survey for England 2006 and Jaqmin-Gadda et al. (2013) and Jaqmin-Gadda et al. (2023) used data from the Paquid cohort, a 22-year long longitudinal study.

#### 3.3.2 Hypertension scenarios investigated

All the studies evaluated hypothetical modifications to hypertension prevalence or incidence which varied from decreases of 10% in hypertension prevalence, [27,28,29] to the complete elimination of hypertension. [30] Lin et al. assessed the effects of a 10% decrease in hypertension prevalence, while Chen et al. (2023) and Jacqmin-Gadda et al. (2013) assessed the effect of decreasing hypertension prevalence by 50%. Jacqmin-Gadda et al. (2013), Jacqmin-Gadda et al. (2023), and Zissimopoulos et al. (2018) assessed the complete disappearance of hypertension. Zissimopoulos et al. (2018) also evaluated the effects of reducing incident hypertension by 50% and having no incident hypertension. Additionally, Jacqmin-Gadda et al. (2013) evaluated the use of antihypertensive drugs that reduce the risk of dementia by 15% and risk of death by 43%, which was the only study that modelled a health intervention in addition to hypothetical changes in prevalence.

Barnes and Yaffe (2011), Luck et al. (2016), and Norton et al. (2014) predicted the prevalence of Alzheimer's using population attributable fractions and adjusted the values for different regions. Barnes and Yaffe (2011) assessed the reduction of hypertension prevalence by 10% or 25%, and Luck et al. (2016) assessed the reduction of hypertension prevalence by 10%, 20% and 50%. Norton et al. (2014) assessed the effect of reducing the prevalence of a combination of risk factors (diabetes mellitus, midlife hypertension, midlife obesity, physical inactivity, depression, smoking, low education) by 10% or 20% per decade.

#### 3.3.3 Findings of partial economic evaluations

Lin et al. (2014) was the only study that evaluated costs, for which only direct health care costs for the United States public health insurance system were considered. The average lifetime per capita cost decreased by Int'l$2783 ($2038 in 2012 USD). In total, a reduction of 10% in hypertension was estimated to save Int'l$32.7 billion ($24 billion in 2012 USD) for the Medicare and Medicaid systems in the United States for the lifetime of 76 million baby boomers.

Generally, reductions in hypertension led to increased life expectancy. Zissimopoulos et al. (2018) estimated that a 50% reduction of hypertension incidence would increase life expectancy by 0.36 years, [30] while Jacqmin-Gadda et al. (2023) predicted a life expectancy increase of 1.52 years for men and 0.96 years for women for the complete disappearance of hypertension. [26]

Several studies did not account for the changes in mortality associated with modifying hypertension prevalence. These studies found that decreases in hypertension led to decreased dementia prevalence. Jacqmin-Gadda et al. (2023) estimated that the complete elimination of hypertension would decrease dementia prevalence by 2.05% for men and 2.04% for women. Barnes and Yaffe (2011) estimated that worldwide, 10% and 25% reductions in hypertension prevalence would lead to decreases of 0.47% (160,000 cases avoided out of 33,900,000) and 1.18% (400,000 cases avoided out of 33,900,000) fewer dementia cases respectively. Similarly, assuming a prevalence of 1 million dementia cases in Germany, Luck et al. (2016) estimated that 10%, 25%, and 50% reductions in hypertension prevalence would lead to a 0.70%, 1.74%, and 3.63% reductions in dementia prevalence respectively. Lastly, Norton et al. (2014) estimated that worldwide, 10% and 20% reductions in the prevalence of a combination of risk factors (diabetes mellitus, midlife hypertension, midlife obesity, physical inactivity, depression, smoking, low education) per decade could lead to a decrease of 8.3% and 15.3% in expected Alzheimer's respectively over 40 years.

Three of the studies considered the impact of decreasing hypertension on mortality rates. Generally, hypertension decreases led to a higher dementia prevalence due to the relative strength of the effect of hypertension status on mortality and dementia risk. Jacqmin-Gadda et al. (2013) evaluated the effect of a hypothetical antihypertensive drug that reduces the risk of dementia by 15% and risk of death by 43%, which led to a 5.6% increase in dementia prevalence after 20 years. Chen et al. (2023) reported that from 2020 to 2060, a 50% decrease in hypertension prevalence would lead to a small increase in dementia prevalence (9.0 cases of dementia per 100,000 people) and an 8% increase in healthy life expectancy at age 65 (5.3 years vs. 4.9 years). Similarly, Zissimopoulos et al. (2018) estimated that a 50% reduction in incident hypertension would lead to 200,000 more dementia cases (11,860,000 vs. 11,660,000 cases) by 2040 due to an increased life expectancy.

Analyses were conducted across different geographic regions and age groups: global (*n* = 2) [23,29], United Kingdom (*n* = 2) [24,29], France (*n* = 2) [25,26], United States (*n* = 4), Germany (*n* = 1) [28], and Europe overall (*n* = 1) [29]. Heterogeneity in study methodology and reported results limits comparability of results within regions. Two studies included cohorts starting at age 65 [25,27] while the remaining studies modeled midlife cohorts (i.e. 45 years onward) . Generally, studies with shorter time horizons and later intervention had smaller intervention effects.

Only 2 studies investigated sex differences in intervention effectiveness. [25,26] They both found stronger intervention effects for men than women, with men experiencing larger increases in life expectancy and dementia risk reduction compared to women.

### 3.4 Quality assessment

The scores from the CHEC-extended checklist varied across all studies. An ideal study would be a full economic evaluation with incremental analysis that clearly outlines the study population and competing alternatives, has a lifetime time horizon and societal perspective, and describes all model assumptions. [31]

Scores for the full economic evaluations ranged from 13 to 20 out of 20 (Table 3). Half of the studies did not report an appropriate time horizon. [21,22] The oldest individuals were aged 80 years and 70 years at the end of the models, which may not encompass lifetime dementia risk. Two of the studies did not perform an incremental analysis, [19,21] discuss the generalizability of the findings [19,21], or discuss ethical and distributional issues appropriately [19,21].

**Table 3:** CHEC Quality Assessment for Full Economic Evaluations (*n* = 4).

Study	CHEC Criteria Number																				
	1	2	3	4	5	6	7	8	9	10	11	12	13	14	15	16	17	18	19	20	Total
**McRae 2021**	Y	Y	Y	Y	N	Y	Y	Y	Y	Y	Y	Y	Y	N	Y	Y	Y	N	Y	N	16
**Mukadam 2020**	Y	Y	Y	Y	Y	Y	Y	Y	Y	Y	Y	Y	Y	Y	Y	Y	Y	Y	Y	Y	20
**Soto-Gordoa 2015**	Y	Y	Y	Y	Y	N	Y	Y	Y	Y	Y	Y	N	N	N	N	Y	N	Y	N	13
**Zhang 2011**	Y	Y	Y	Y	Y	N	Y	Y	Y	Y	Y	Y	Y	Y	Y	Y	Y	Y	Y	Y	19
**Total**	4	4	4	4	3	2	4	4	4	4	4	4	3	2	3	3	3	2	4	2	

The partial economic evaluations satisfied fewer CHEC criteria compared to the full economic evaluations (Table 4). Scores for the partial economic evaluations ranged from 6 to 13 out of 20. Studies with higher scores clearly reported their target population (5/8), discussed ethical and distributional issues appropriately (5/8), discussed the generalizability of their results to other settings (4/8), and conducted sensitivity analysis (2/8).

**Table 4:** CHEC Quality Assessment for Partial Economic Evaluations (*n* = 8).

Study	CHEC Criteria Number																				
	1	2	3	4	5	6	7	8	9	10	11	12	13	14	15	16	17	18	19	20	Total
**Barnes and Yaffe 2011**	N	Y	Y	N	Y	N	N	N	N	N	Y	Y	Y	N	N	N	Y	Y	Y	Y	10
**Chen 2023**	Y	Y	Y	N	Y	Y	N	N	N	N	Y	Y	Y	N	N	Y	Y	Y	Y	N	12
**Jacqmin-Gadda 2013**	Y	Y	Y	N	Y	Y	N	N	N	N	Y	Y	Y	N	N	Y	Y	Y	N	Y	11
**Jacqmin-Gadda 2023**	Y	Y	Y	N	Y	Y	N	N	N	N	Y	Y	Y	N	N	N	Y	N	Y	N	10
**Lin 2014**	Y	Y	Y	Y	Y	Y	N	N	Y	Y	Y	Y	Y	N	N	N	Y	N	N	Y	13
**Luck 2016**	N	Y	Y	N	N	N	N	N	N	N	Y	Y	N	N	N	N	Y	N	Y	N	6
**Norton 2014**	N	Y	N	N	Y	N	N	N	N	N	Y	Y	Y	N	N	N	Y	Y	Y	Y	9
**Zissimopoulos 2018**	Y	Y	Y	N	Y	Y	N	N	N	N	Y	Y	N	N	N	N	Y	N	Y	Y	10
**Total**	5	8	7	1	7	6	0	0	1	1	8	8	6	0	0	2	8	4	6	5	

## 4 Discussion

The present systematic review found 12 studies evaluating the effects of modifying hypertension on the economic burden of dementia. The review found four full economic evaluations and eight partial economic evaluations, with one evaluating cost and seven modelling prevalence only. Only three studies [20,22,25] evaluated the implementation of a potential applied intervention. Overall, there were very heterogeneous methodologies and differing study types, especially among the full economic evaluations.

Previous reviews of dementia interventions have indicated potential cost-effectiveness of dementia prevention. A previous scoping review of full economic evaluations of primary prevention interventions for dementia found 7 studies targeting a range of dementia risk factors. The ICERs for these studies ranged from -Int'l$ 909,864 (−80,427.97 Euros) (intervention dominated) to Int'l$117,869 (104,189.82 Euros) per QALY. [32] Another systematic review of cost-effectiveness studies of community and population-based interventions to reduce dementia by Walsh et al. (2022) found 45 studies, which targeted a variety of factors, including smoking (*n* = 15), education (*n* = 10), and physical activity (*n* = 9). [33] Overall, interventions were cost effective.

Overall, there is a lack of full economic evaluations that evaluate implemented hypertension interventions and its impact on dementia. This may be partly due to limitations such as the long time between hypertension, typically occurring in mid-life, and the onset of dementia which may occur decades later. However, many observational studies have shown strong evidence for the introduction of interventions such as hypertension treatment. A recent meta-analysis of 17 studies found that those with uncontrolled hypertension had 42% higher risk of dementia compared to control without dementia and those with treated hypertension. [34] There was no significant difference in risk between those without hypertension and those who received treatment for hypertension. In addition, a randomized controlled trial of an improved blood pressure lowering intervention decreased dementia risk by 15% compared to usual care. [35] Another trial found that intensive blood pressure control reduced the risk of mild cognitive impairment and probable dementia by 19%. A previous full economic evaluation assessed the impact of a national diabetes intervention on dementia burden in the UK, [36] demonstrating the possibility of this type of intervention being applied to dementia prevention.

All studies included in the review were conducted from the perspective of high-income countries. The studies that included estimates on a global level [23,29] were prevalence studies that used general global rates of risk factors to provide estimates and did not have any specific data on any low-or middle-income countries (LMICs). In addition, they only conducted subgroup analysis for the United States (*n* = 2), United Kingdom (*n* = 1), and Europe (*n* = 1). It is estimated that 61% of people with dementia live in LMICs. [37] The WHO global plan to respond to dementia highlights the need to improve dementia risk reduction on a global scale. In addition, globally, only 42% of adults with hypertension are diagnosed and treated and 46% of adults with hypertension are unaware they have it. [13] In low- and middle-income countries, only a third of people are aware of their hypertension status and around 8% of those with hypertension have their blood pressure controlled. [38] Due to the low baseline hypertension care coverage, interventions such as hypertension screening and treatment have the potential to be especially impactful in these areas.

There is also a general lack of economic studies for dementia prevention interventions in LMICs. A recent updated review of model-based economic evaluations of dementia interventions found three studies that evaluated primary prevention. [39] The studies had differing interventions and methodologies, and none were from LMICs. Walsh et al. (2022) included studies from LMICs, though none of the ten studies explicitly modelled dementia or considered dementia risk reduction as a goal of their interventions. The lack of economic evidence particularly evaluating the cost-effectiveness of hypertension interventions in LMICs is an area that requires further study.

None of the studies in the review differentiated between vascular dementia and other types of dementia such as Alzheimer's. The relationship between hypertension, stroke, and vascular dementia is well studied [7,26,40] but the evidence for the connection between hypertension and other dementias such as Alzheimer's disease is less robust. [40] Given the different risk factors between Alzheimer's disease and vascular dementia, future studies should consider modelling the impact of hypertension interventions on vascular dementia explicitly.

Most economic models initiated cohorts between the ages of 45 and 64, though some began at age 65. The starting age of the cohort can significantly influence cost-effectiveness outcomes. Hypertension interventions targeting older populations may yield diminished health benefits in terms of dementia risk reduction. Moreover, models that begin at an older age may underestimate both the costs and benefits of hypertension interventions.

The association between hypertension via the increased risk for stroke is well studied and it is estimated that 55% of the risk of dementia owing to hypertension can be attributed to stroke. [41] However, only one study in the review considered the effect of stroke on dementia burden. [30] In addition, the CAIDE dementia risk score used in two of the studies [21,22] did not consider increased stroke risk. There is currently no full economic evaluation that also models the decreased risk of stroke associated with decreased hypertension and the resulting reduction in dementia risk.

To adequately assess the economic impact of an intervention, full economic evaluations are preferred as it can provide insight on cost effectiveness, however most of the literature identified in this review consisted of partial economic evaluations. The existing full economic evaluations have very heterogeneous methodologies and only two assessed potential applied health interventions. In addition, the studies that used antihypertensive treatment as a predictor for hypertension control assumed all those who were treated were able to achieve hypertension control; this does not consider treatment resistant cases and those who might not adhere to treatment. Moreover, none of the identified studies considered the real-world effectiveness of hypertension modification interventions, indicating a large gap in the literature.

One of the largest studies of hypertension control and dementia, i.e. the SPRINT trial, evaluated the effectiveness of intensive blood pressure control (<120 mmHg) to standard blood pressure control (<140 mmHg). The trial found a 20% reduced risk of mild cognitive impairment for those receiving intensive blood pressure treatment but did not find significant decreases in dementia. [42] However, the trial included those aged 50 and older with a mean age of 67.9 years and followed them for an average length of 5 years. Given that the incidence of dementia increases with age, with one study estimating the mean age of onset to be close to 80 years [43], it is plausible that the follow-up period may not be long enough to observe a significant difference in the rate of dementia. In addition, interventions applied to late-life hypertension tend to have a weaker association with dementia than midlife hypertension management. [44] The SPIRIT trial ended early due to improvements seen in cardiovascular outcomes, but a subsequent study is following patients for longer to see the true effect of blood pressure control on dementia. [45] As more research emerges on the relationship between blood pressure levels and dementia, the economic evidence will need to be updated.There are several additional limitations of these full economic evaluations. McRae et al. (2021) parametrised their model using wide estimates rather than values from specific studies, impacting the precision of the estimates. Soto-Gordoa et al. (2015) did not consider the intervention costs and did not conduct incremental analyses in their results. In addition, Mukadam et al. (2020), used utility values derived from two different assessment tools. Mild and moderate dementia used EQ-5D, whereas the severe stage used the Health Utilities Index Mark II (HUI2). The utility values for mild, moderate, and severe dementia stages are 0.714, 0.64, and 0.385, respectively. In general, HUI scores tend to be lower than EQ-5D scores and the difference in assessment tool may impact the final estimates. [46,47] However, in the sensitivity analyses, the parameter values for severe dementia did not impact estimates as much as other parameters. For Zhang et al. (2011), the intervention evaluated was a combination intervention where the individual effect of hypertensive treatment was not assessed. Overall, there remains a gap in the literature for full economic evaluations that assess practical health services hypertension interventions and its effect on dementia burden.

The present study has several limitations. The studies in the present review showed heterogeneity in the interventions, particularly in full economic evaluations, which limited the ability to synthesise the results. The diversity of hypertension modification programs made meta-analysis or formal quantitative synthesis of the evidence infeasible. Despite the heterogeneity in the interventions examined, the economic evidence indicated the potential cost-effectiveness of these approaches. However, the extent of economic value varies across different interventions.

The present review included interventions or modification programs specifically designed to reduce hypertension. Interventions that reduced hypertension as a secondary objective, such as the implementation of a physical activity program, were not included in the review if modifying blood pressure, and subsequently decreasing the risk of dementia was not explicitly stated as a goal of the intervention. Due to this, the body of literature for interventions that may prevent dementia through modifying hypertension as a risk factor may be larger than what was included in the review. In addition, interventions that modify the risk of stroke to prevent dementia were not included if they did not explicitly include modifying or preventing hypertension. Modifying stroke can happen through many different risk pathways, one of which is hypertension. These studies were not included as it would be difficult to distinguish the effect of the hypertension intervention and stroke effects due to the high degree of interconnectedness. Lastly, hypertension is a risk factor for many cardiovascular events, therefore the economic impact of modifying hypertension is likely underestimated in the included studies, as other cardiovascular events were not modelled.

## 5 Conclusion

Overall, hypothetical decreases in hypertension prevalence were associated with reduction in dementia rates, assuming that mortality rates remained unchanged. With changes in mortality considered, hypertension reduction led to a higher lifetime risk for dementia due to a longer life expectancy. Effective control and prevention of hypertension can potentially be a cost-effective strategy for reducing the dementia burden with the added benefits of reducing the risks and burden of other conditions such as stroke and heart disease. However, the scarcity and heterogeneity of evidence makes inference difficult and highlights the need for further research and economic evaluations of applied interventions. In addition, future studies should explicitly consider stroke in the risk and cost estimates.

## Funding

Funding for this research was provided by the Weston Family Foundation through the 10.13039/100012479Weston Brain Institute (grant TR202092). M. Lan has received the Ontario Graduate Scholarship (Government of Ontario). S. Ali and K.K. Anderson are partly funded through the Canada Research Chair program (Government of Canada). The sponsors had no role in the design and conduct of the study; in the collection, analysis, and interpretation of data; in the preparation of the manuscript; or in the review or approval of the manuscript.

## Declaration of competing interest

M. Lan reports grants from the Ontario Graduate Scholarship Program, during the conduct of the study. S. Ali reports grants from the Weston Brain Institute (Grant TR202092), during the conduct of the study. A. John-Baptiste, C. Curran, F.W. Alemu, A. Avan, and K.K. Anderson have nothing to disclose. The sponsors had no role in the design and conduct of the study; in the collection, analysis, and interpretation of data; in the preparation of the manuscript; or in the review or approval of the manuscript.

## Appendix

### A.1 Search Strategies

*MEDLINE*

1 Economics/
2 exp "Costs and Cost Analysis"/
3 Economics, Nursing/
4 Economics, Nursing/
5 Economics, Pharmaceutical/
6 exp Economics, Hospital/
7 Economics, Dental/
8 exp "Fees and Charges"/
9 exp Budgets/
10 budget*.ti,ab,kf.
11 (economic* or cost or costs or costly or costing or price or prices or pricing or pharmacoeconomic* or pharmaco-economic* or expenditure or expenditures or expense or expenses or financial or finance or finances or financed).ti,kf.
12 (economic* or cost or costs or costly or costing or price or prices or pricing or pharmacoeconomic* or pharmaco-economic* or expenditure or expenditures or expense or expenses or financial or finance or finances or financed).ab. /freq=2
13 (cost* adj2 (effective* or utilit* or benefit* or minimi* or analy* or outcome or outcomes)).ab,kf.
14 (value adj2 (money or monetary)).ti,ab,kf.
15 exp models, economic/
16 economic model*.ab,kf.
17 markov chains/
18 markov.ti,ab,kf.
19 monte carlo method/
20 exp Decision Theory/
21 (decision* adj2 (tree* or analy* or model*)).ti,ab,kf.
22 exp "Health Care Economics and Organizations"/ or exp "Cost of Illness"/
23 (Economic Evaluation or Health economics or Cost-effectiveness or Cost-benefit or Cost analysis or Cost saving or Cost control or Cost utility or Cost per qaly or Economic analysis or cost or cost* or Economics or Healthcare cost or Health care cost or Markov or Markov model or Decision analyses or Decision analysis or Decision tree or Decision model or Cost consequence).tw,kf.
24 exp Dementia, Vascular/ or exp Dementia/ or exp "Mental Status and Dementia Tests"/ or exp Frontotemporal Dementia/ or exp Dementia, Multi-Infarct/
25 exp Cognitive Dysfunction/ or exp Alzheimer Disease/
26 (Dementia or Alzheimers or dement* or alzheimer* or cognitive impairment or (cogniti* adj2 impair*) or binswanger or binswang*).tw,kf.
27 Intracranial Hypertension/ or Hypertension, Pulmonary/ or Familial Primary Pulmonary Hypertension/ or White Coat Hypertension/ or Essential Hypertension/ or Sinistral Portal Hypertension/ or Dietary Approaches To Stop Hypertension/ or Ocular Hypertension/ or Isolated Systolic Hypertension/ or Hypertension, Pregnancy-Induced/ or Hypertension/ or Hypertension, Renovascular/ or Masked Hypertension/ or Pulmonary Arterial Hypertension/ or Idiopathic Noncirrhotic Portal Hypertension/ or Hypertension, Renal/ or Hypertension, Malignant/ or Intra-Abdominal Hypertension/ or Hypertension, Portal/
28 exp Hypertension/
29 exp Blood Pressure/
30 (hypertension or hypertens* or blood pressure or high blood pressure or elevated blood pressure or prehypertension or prehyperten* or ((arterial or diastolic or systolic) adj2 pressure) or ((arterial or diastolic or systolic) adj pressure)).tw,kf.
31 exp Essential Hypertension/
32 1 or 2 or 3 or 4 or 5 or 6 or 7 or 8 or 9 or 10 or 11 or 12 or 13 or 14 or 15 or 16 or 17 or 18 or 19 or 20 or 21 or 22 or 23
33 24 or 25 or 26
34 27 or 28 or 29 or 30 or 31
35 32 and 33 and 34

*EMBASE*

1 Economics/
2 Cost/
3 exp Health Economics/
4 Budget/
5 budget*.ti,ab,kf.
6 (economic* or cost or costs or costly or costing or price or prices or pricing or pharmacoeconomic* or pharmaco-economic* or expenditure or expenditures or expense or expenses or financial or finance or finances or financed).ti,kf.
7 (economic* or cost or costs or costly or costing or price or prices or pricing or pharmacoeconomic* or pharmaco-economic* or expenditure or expenditures or expense or expenses or financial or finance or finances or financed).ab. /freq=2
8 (cost* adj2 (effective* or utilit* or benefit* or minimi* or analy* or outcome or outcomes)).ab,kf.
9 (value adj2 (money or monetary)).ti,ab,kf.
10 Statistical Model/
11 exp economic model/
12 economic model*.ab,kf.
13 Probability/
14 markov.ti,ab,kf.
15 monte carlo method/
16 monte carlo.ti,ab,kf.
17 Decision Theory/
18 Decision Tree/
19 (decision* adj2 (tree* or analy* or model*)).ti,ab,kf.
20 exp economic evaluation/ or exp "cost of illness"/
21 (Economic Evaluation or Health economics or Cost-effectiveness or Cost-benefit or Cost analysis or Cost saving or Cost control or Cost utility or Cost per qaly or Economic analysis or cost or cost* or Economics or Healthcare cost or Health care cost or Markov or Markov model or Decision analyses or Decision analysis or Decision tree or Decision model or Cost consequence).tw,kf.
22 exp Alzheimer disease/
23 exp cognitive defect/
24 semantic dementia/ or exp mixed dementia/ or dementia assessment/ or exp dementia/ or clinical dementia rating scale/ or Cornell Scale for Depression in Dementia/ or Mattis Dementia Rating Scale/ or frontal variant frontotemporal dementia/ or Pick presenile dementia/ or Blessed dementia scale/ or senile dementia/ or "mixed depression and dementia"/ or multiinfarct dementia/ or frontotemporal dementia/ or presenile dementia/ or HIV associated dementia/
25 (Dementia or Alzheimers or dement* or alzheimer* or cognitive impairment or (cogniti* adj2 impair*) or binswanger or binswang*).tw,kf.
26 orthostatic hypertension/ or portopulmonary hypertension/ or idiopathic noncirrhotic portal hypertension/ or hypoxia-induced pulmonary hypertension/ or experimental hypertension/ or deoxycorticosterone-salt-induced hypertension/ or pulmonary hypertension/ or chronic thromboembolic pulmonary hypertension/ or renovascular hypertension/ or l-NAME-induced hypertension/ or portal hypertension/ or hereditary hypertension/ or diabetic hypertension/ or hypertension encephalopathy/ or systolic hypertension/ or monocrotaline-induced pulmonary hypertension/ or white coat hypertension/ or intracranial hypertension/ or non-cirrhotic portal hypertension/ or experimental renovascular hypertension/ or intraocular hypertension/ or masked hypertension/ or borderline hypertension/ or maternal hypertension/ or two-kidney, one-clip renovascular hypertension/ or isolated systolic hypertension/ or hypertension retinopathy/ or experimental portal hypertension/ or essential hypertension/ or intraabdominal hypertension/ or malignant hypertension/ or persistent pulmonary hypertension/ or idiopathic intracranial hypertension/ or experimental pulmonary hypertension/ or sinistral portal hypertension/ or resistant hypertension/ or exp hypertension/
27 exp blood pressure/
28 Hyp
29 exp essential hypertension/
30 1 or 2 or 3 or 4 or 5 or 6 or 7 or 8 or 9 or 10 or 11 or 12 or 13 or 14 or 15 or 16 or 17 or 18 or 19 or 20 or 21
31 22 or 23 or 24 or 25
32 26 or 27 or 28 or 29
33 30 and 31 and 32

*SCOPUS*

(TITLE-ABS-KEY (economic* OR cost OR costs OR costly OR costing OR price OR prices OR pricing OR pharmacoeconomic* OR pharmaco-economic* OR expenditure OR expenditures OR expense OR expenses OR financial OR finance OR finances OR financed OR "value of money" OR "Economic Evaluation" OR "Health economics" OR "Cost-effectiveness" OR "Cost-benefit" OR "Cost analysis" OR "Cost saving" OR "Cost control" OR "Cost utility" OR "Cost per qaly" OR "Economic analysis" OR cost OR cost* OR economics OR "Healthcare cost" OR "Health care cost" OR markov OR "Markov model" OR "Decision analyses" OR "Decision analysis" OR "Decision tree" OR "Decision model" OR "Cost consequence") AND TITLE-ABS-KEY (dementia OR alzheimers OR dement* OR alzheimer* OR "cognitive impairment" OR binswanger OR binswang*) AND TITLE-ABS-KEY (hypertension OR hypertens* OR "blood pressure" OR "high blood pressure" OR "elevated blood pressure" OR prehyperten* OR arterial OR diastolic OR systolic))

*WEB OF SCIENCE*

(TS= (economic* OR cost OR costs OR costly OR costing OR price OR prices OR pricing OR pharmacoeconomic* OR pharmaco-economic* OR expenditure OR expenditures OR expense OR expenses OR financial OR finance OR finances OR financed OR "value of money" OR "Economic Evaluation" OR "Health economics" OR "Cost-effectiveness" OR "Cost-benefit" OR "Cost analysis" OR "Cost saving" OR "Cost control" OR "Cost utility" OR "Cost per qaly" OR "Economic analysis" OR cost OR cost* OR economics OR "Healthcare cost" OR "Health care cost" OR markov OR "Markov model" OR "Decision analyses" OR "Decision analysis" OR "Decision tree" OR "Decision model" OR "Cost consequence") AND TS=(dementia OR alzheimers OR dement* OR alzheimer* OR "cognitive impairment" OR binswanger OR binswang*) AND TS= (hypertension OR hypertens* OR "blood pressure" OR "high blood pressure" OR "elevated blood pressure" OR prehyperten* OR arterial OR diastolic OR systolic))

### A.2 CHEC-extended checklist items

1. Is the study population clearly described?
2. Are competing alternatives clearly described
3. Is a well-defined research question posed in answerable form?
4. Is the economic study design appropriate to the stated objective?
5. Are the structural assumptions and the validation methods of the model properly reported?
6. Is the chosen time horizon appropriate in order to include relevant costs and consequences?
7. Is the actual perspective chosen appropriate?
8. Are all important and relevant costs for each alternative identified?
9. Are all costs measured appropriately in physical units?
10. Are costs valued appropriately?
11. Are all important and relevant outcomes for each alternative identified?
12. Are all outcomes measured appropriately?
13. Are outcomes valued appropriately?
14. Is an appropriate incremental analysis of costs and outcomes of alternatives performed?
15. Are all future costs and outcomes discounted appropriately?
16. Are all important variables, whose values are uncertain, appropriately subjected to sensitivity analysis?
17. Do the conclusions follow from the data reported?
18. Does the study discuss the generalizability of the results to other settings and patient/client groups?
19. Does the article/report indicate that there is no potential conflict of interest of study researcher(s) and funder(s)?
20. Are ethical and distributional issues discussed appropriately?
